# Lanatoside C, a cardiac glycoside, acts through protein kinase Cδ to cause apoptosis of human hepatocellular carcinoma cells

**DOI:** 10.1038/srep46134

**Published:** 2017-04-07

**Authors:** Min-Wu Chao, Tzu-Hsuan Chen, Han-Li Huang, Yu-Wei Chang, Wei-Chun HuangFu, Yu-Ching Lee, Che-Ming Teng, Shiow-Lin Pan

**Affiliations:** 1 The Ph.D. Program for Cancer Biology and Drug Discovery, College of Medical Science and Technology, Taipei Medical University, Taipei, Taiwan; 2 Division of Industrial Promotion, Development Center for Biotechnology, Taipei, Taiwan; 3 Pharmacological Institute, College of Medicine, National Taiwan University, Taipei, Taiwan; 4 The Center of Translational Medicine, Taipei Medical University, Taipei, Taiwan; 5 Ph.D. Program for Biotechnology in Medicine, College of Medical Science and Technology, Taipei Medical University, Taipei, Taiwan; 6 School of Pharmacy, College of Pharmacy, Taipei Medical University, Taipei, Taiwan; 7 Department of Pharmacology, College of Medicine, Taipei Medical University, Taipei, Taiwan.

## Abstract

Recent studies have revealed that cardiac glycosides, such as digitalis and digoxin, have anticancer activity and may serve as lead compounds for the development of cancer treatments. The poor prognosis of hepatocellular carcinoma (HCC) patients reflects the development of resistance to current chemotherapeutic agents, highlighting the need for discovering new small-molecule therapeutics. Here, we found that lanatoside C, an anti-arrhythmic agent extracted from *Digitalis lanata*, inhibited the growth of HCC cells and dramatically decreased tumor volume as well as delayed tumor growth without obvious body weight loss. Moreover, lanatoside C triggered mitochondrial membrane potential (MMP) loss, activation of caspases and translocation of apoptosis-inducing factor (AIF) into the nucleus, which suggests that lanatoside C induced apoptosis through both caspase-dependent and -independent pathways. Furthermore, we discovered that lanatoside C activated protein kinase delta (PKCδ) via Thr505 phosphorylation and subsequent membrane translocation. Inhibition of PKCδ reversed lanatoside C-induced MMP loss and apoptosis, confirming that lanatoside C caused apoptosis through PKCδ activation. We also found that the AKT/mTOR pathway was negatively regulated by lanatoside C through PKCδ activation. In conclusion, we provide the first demonstration that the anticancer effects of lanatoside C are mainly attributable to PKCδ activation.

Hepatocellular carcinoma (HCC), the most common type of primary liver cancer, is the fifth-leading cause of cancer-related death worldwide^1^. The incidence of HCC is not uniformly distributed geographically, with the highest frequency found in developing countries^2^. HCC often results from chronic liver diseases that accompany hepatitis B or C infection. Clinical management options for HCC, including surgical resection, liver transplantation, percutaneous ethanol injection, radiofrequency ablation, transarterial embolization as well as systemic chemotherapy, depend on tumor staging^3^. Unfortunately, HCC exhibits a high recurrence rate and relatively poor overall survival owing to late-stage disease at presentation, liver dysfunction, cellular resistance to conventional cytotoxic agents, and ineffectiveness of drugs^4^. Thus, developing an effective therapeutic strategy for HCC patients with an advanced form of the disease has always been a crucial issue.

Cardiac glycosides comprise a large family of naturally derived compounds that share a common structural motif. Their core structure consists of a steroidal framework, which is considered the pharmacophoric moiety responsible for the activity of these compounds^5^. Lanatoside C and digoxin are structurally closely related in that digoxin can be obtained from lanatoside C by hydrolytic removal of the acetyl and glucose moieties (Fig. 1A)^6^. The major pharmacological effect of cardiac glycosides, still widely used clinically, is inhibition of the plasma membrane Na^+^/K^+^ ATPase, which indirectly enhances cardiac muscle contractile force. However, recent studies suggest that several cardiac glycosides has been reported to exert anticancer activity through different mechanisms, such as the SRC/EGFR/RAS/ERK signaling pathway, p21, NF-κB, AP-1, topoisomerase and HIF-1, some of which do not involve targeting the Na^+^/K^+^ ion pump^7^,^8^,^9^,^10^,^11^,^12^. These observations suggest that cardiac glycosides could be repurposed as promising anticancer candidates.

The mammalian protein kinase C (PKC) family of serine/threonine kinases is classified into three subfamilies based on calcium dependence and activators: conventional PKC (cPKC), novel PKC (nPKC), and atypical PKC (aPKC). PKC acts through a number of pathways to regulate the expression of genes that control cell survival, apoptosis, tumorigenesis, and/or metastasis. Diverse PKC isoenzymes act via complex mechanisms to exert variable responses in different cell types^13^. PKCδ, a member of the nPKC subfamily, was first isolated from the particulate fraction of mouse epidermis^14^. It has been reported that PKCδ activation, triggered by translocation to the cell membrane, is involved in many essential cellular processes, including regulation of cell growth and apoptosis in cells exposed to stimuli such as Fas, ultraviolet (UV) radiation, or etoposide-class chemotherapeutic drugs. In addition, PKCδ is proteolytically activated by caspase-3. Subsequent translocation of the cleaved catalytic domain to mitochondria or the nucleus leads to mitochondrial membrane potential (MMP) loss and ultimately elicits apoptosis^15^. As a consequence of its crucial involvement in cell death mechanisms, PKCδ is a potential therapeutic target for anticancer therapy.

Cardiac glycosides, such as digoxin, digitoxin and ouabain, have been used clinically to treat congestive heart failure and anti-arrhythmias. Nevertheless, numerous studies have revealed beneficial effects of these compounds in cancer treatment. In this study, we examined the antitumor activity of lanatoside C in human HCC cells and assessed its mechanism of action, providing the first demonstration that PKCδ is a key mediator of lanatoside C-induced apoptosis.

## Materials and Methods

### Reagents

Lanatoside C (lana.C), rottlerin, Ro318220, Gö6983 were purchased from Sigma Chemical Co. (St Louis, MO, USA). The above drugs were all dissolved in dimethylsulfoxide (DMSO). The non-conjugated primary antibodies were against C23 (nucleolin, Sigma Chemical Co., St Louis, USA), BID, caspase-8, caspase-9, PKCδ (Thr505), PKCδ, caveolin, phoshpo-p44/42 MAP kinas (Thr202/Tyr204), p44/42 MAP kinas (Thr202/Tyr204), phosho-SAPK/JNK (Thr183/Tyr185), p38 MAP kinase, phosho-mTOR, phosphor-p70 S6 kinase (Thr421/Ser424), phosphor-4E-BP1 (Thr37/46), phosho-eIF4E (Ser209), AKT (Cell signaling Technology, Beverly, MA, USA), AIF, Bcl-xl, Mcl-1, α-tubulin, PARP-1/2 (Santa Cruz Biotechnology Corp., Santa Cruz, CA, USA), caspase-2, caspase-6, caspase-7 (BD Biosciences, San Jose, CA, USA), phosho-AKT (Ser473, Epitomics Inc., Burlingame, CA, USA), caspase-3 (Imgenex, San Diego, CA, USA), β-actin (Millipore, Temecula, CA, USA). The secondary horseradish peroxidase (HRP)-conjugated anti-mouse and anti-rabbit IgGs were purchased from Santa Cruz Biotechnology (Santa Cruz, CA, USA). z-VAD-fmk was purchased from R&D system (Minneapolis, MN, USA).

### Cell culture

Human hepatocellular carcinoma cell lines, Hep3B and HA22T, were purchased from Bioresource Collection and Research Center (Hsinchu, Taiwan) with p127 and p57, respectively. The cells were cultured in RMPI-1640 supplemented with 10% FBS (fetal bovine serum), L-gultamine (2 μM) and antibiotics (penicillin, streptomycin and amphotericin B) and incubated at 37 °C in a humidified atmosphere of 5% CO_2._

### Cell proliferation assay

Cells were seeded in 96-well plates (3,500 cells/well) overnight for attachment, treated with the indicated concentrations of lanatoside C for 48 h, then were fixed with 10% TCA (trichloroacetic acid), stained with SRB (sulforhodamine B, 0.4% in 1% acetic acid) for 15 min, and then washed repeatedly with 1% acetic acid. SRB dye was purchased from Sigma Chemical Co. (St Louis, MO, USA). The dye was dissolved with trizma base (10 mM) and then measured by ELISA reader at 510 nm. GI_50_ was represented the concentration of 50% growth inhibition.

### Cell viability assay

Cells were treated with the test compound for the indicated time, and then 0.5 mg/ml of MTT (3-(4,5-Dimethylthiazol-2-yl)-2,5-diphenyltetrazolium bromide) solution was added. MTT powder was purchased from Sigma Chemical Co. (St Louis, MO, USA). The plates were incubated at 37 °C for 1 h and the dye was dissolved in DMSO (dimethyl sulfoxide). The absorbance was measured by ELISA reader at 550 nm.

### Terminal deoxynucleotidyl transferase dUTP nick end labeling (TUNEL) assay

TUNEL apoptosis detection kit was purchased from Promega (Madiison, WI, USA). The cells were seeded in a chamber slide and incubated overnight for attachment, and then treated with the indicated drugs. The slide was washed twice with cold PBS, and the cells were fixed with cold methanol for 10 min, and then added with equilibration buffer for 10 min. The supernatant was removed, and TdT (terminal deoxynucleotidyl transferase) incubation buffer (equilibration buffer, nucleotide mix and TdT enzyme) was added into the slide in the dark for 1 h, and the response was terminated by SSC solution. The detection of DNA fragments was observed by fluorescence microscopy under 200 magnifications.

### Cell cycle distribution

Cell cycle distribution was determined by flow cytometry. After cells were treated with lanatoside C, the cells were trypinized and resuspended in 70% cold ethanol at −20 °C for 30 min. Then the cells were washed with PBS, extracted with DNA extraction solution (0.2 M Na_2_HPO_4_ and 0.1 M citric acid buffer) for 15 min, and stained with PI solution (1% Triton X-100, 100 μg/ml RNase and 80 μg/ml propidium iodide in PBS) in the dark for 30 min. Finally, the cells were analyzed by FACScan and CellQuest software.

### *In vivo* xenograft model

Hep3B xenograft model was used to evaluate *in vivo* efficacy of lanatoside C. Hep3B cells (10^6^/mice) were subcutaneously injected into severe combined immunodeficient (SCID) mice. When the average tumor size reached 100 mm^3^, mice were grouped and administered with 2.5 mg/kg lanatoside C in 50% Cremophor EL and 50% DMSO. Body weights and tumor sizes were measured twice a week. Once the average tumor size was greater than 2,500 mm^3^, the mice were sacrificed. Tumor size was calculated by caliper measurement (mm) and ellipsoid sphere formula (LW^2^/2, L: length; W: width). All animal experiments were carried out in accordance with guidelines and regulations followed ethical standards and protocols approved by Animal Use and Management Committee of Taipei Medical University.

### Mitochondrial membrane potential measurement

Mitochondrial membrane potential of cells was monitored by the retention of rhodamine 123 dye (Sigma Chemical Co., St Louis, MO, USA). Cells were incubated with rhodamine 123 for 30 min before harvesting. Then the cells were trypsinized, resuspended in PBS and analyzed by flow cytometry.

### Western blot analysis

Treated cells were incubated for 25 min at 4 °C in lysis buffer (20 mM Tris-HCl buffer, 0.5 mM EGTA, 2 mM EDTA, 2 mM dithiothreitol, 0.5 mM phenymethylsulfonyl fluoride, 10 μg/ml leupeptin) and centrifuged at 13,000 rpm for 15 min at 4 °C. For cytosol and nuclear protein extraction, cells were harvested and lysised in buffer A (pH 7.9 10 mM Hepes, 10 mM KCl, 1.5 mM MgCl_2_, 0.2 mM PMSF and 0.5 mM DTT) for 10 min at 4 °C. The cells were then centrifuged at 3,000 rpm for 15 min. The supernatant was cytosol protein extraction. The remained pellets were incubated in buffer C (pH 7.9 20 mM Hepes, 420 mM NaCl, 25% glycerol, 1.5 mM MgCl_2_, 0.2 mM EDTA, 0.2 mM PMSF and 0.5 mM DTT) for 20 min at 4 °C and centrifuged. The supernatant was nuclear protein. To separate cell cytosol and membrane protein, FractionPREP^TM^ cell Fractionation kit (Biovision, Inc.) was used. Following the protocol, cells were collected and lysised in Cytosol Extraction Buffer-Mix (Cytosol Extraction Buffer, Protease inhibitor Cocktail and DTT) for 20 min on ice then centrifuged at 700 g for 10 min to acquire cytosol extraction. Membrane Extraction Buffer-A Mix (Membrane Extraction Buffer-A, Protease inhibitor Cocktail and DTT) was added in the remaining pellets and the mixtures were vortexed for 10–15 sec repeatedly. Then the mixtures were vortexed in Membrane Extraction Buffer-B for 5 sec on ice. The supernatant was cell membrane extraction after the mixtures were centrifuged at 1000 g for 5 min. Total protein was determined and quantified by using BCA^TM^ Protein Assay Kit (Pierce, Rockford, IL, USA). Equal amounts of protein were separated by SDS-PAGE (sodium dodecyl sulfate polyacrylamide gels) and transferred onto a PVDF (polyvinylidiene difluoride) membrane, which was then blocked by incubation for 1 h at room temperature with 5% fat-free milk in PBS. The membrane was incubated in primary antibodies overnight at 4 °C, followed by in the corresponding HRP-conjugated secondary antibodies for 1 h at room temperature. Bound antibodies were detected using enhanced chemiluminescence (ECL) detection reagents (Advansta Corp., Menlo Park, CA, USA).

### Transfection

Cells were transfected with lipofetamine 2000 (Thermo Fisher Scientific Inc. Carlsbad, CA, USA) in OPTI-MEM medium according to the manufacturer’s protocol. Myr-Akt plasmid and constitutively active MEK plasmid were gifts from Dr. Lin, Chien-Huang (Taipei Medical University, Taiwan) and Prof. P.P. Pandolfi (Harvard Medical School, Boston, MA, USA) respectively. PKCδ siRNA was purchased from Invitrogen (Thermo Fisher Scientific Inc. Carlsbad, CA, USA).

### Statistic anaylsis

All experimental results are repeated with at least three independent experimental replications and expressed as the mean ± SD. The statistical analysis was determined using Bonferroni *t*-test and *P*-values. A *P* value of less than 0.05 was considered statistically significant.

## Results

### Effects of lanatoside C on the proliferation and cell cycle of HCC cell lines

**Figure 1:**
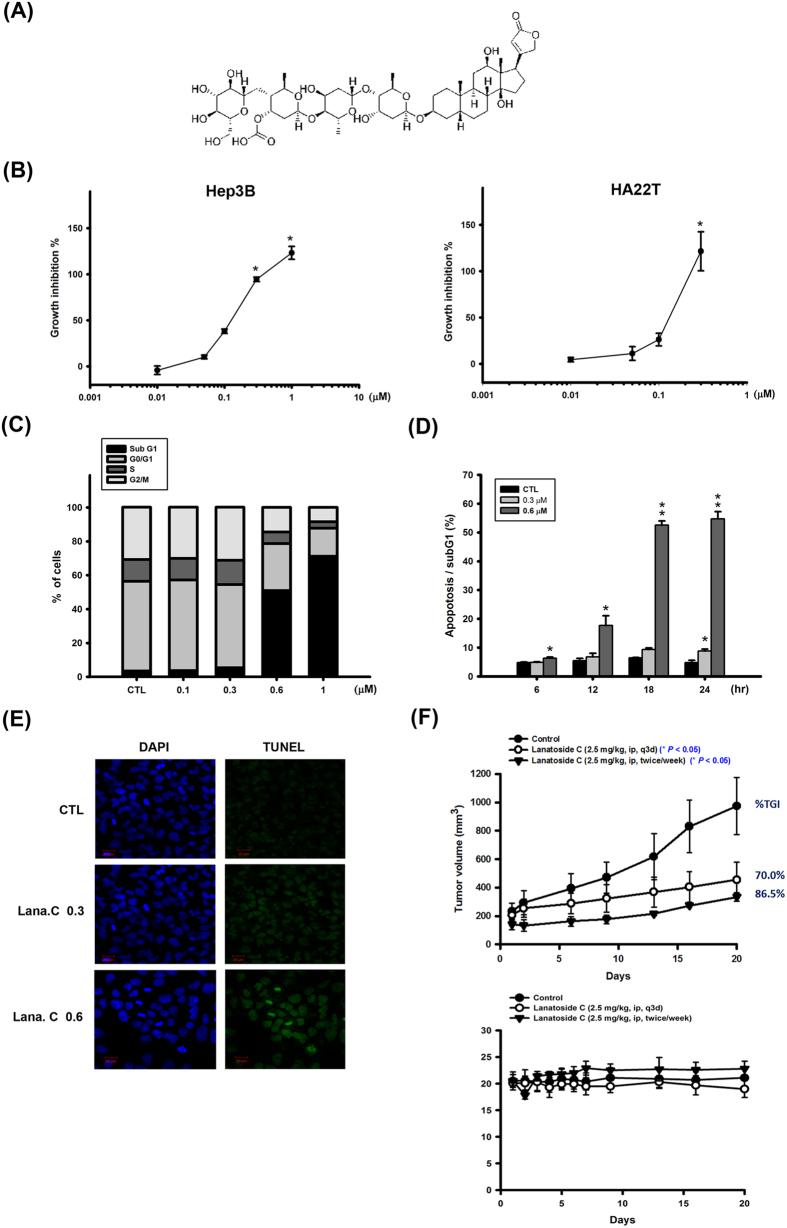
Effect of lanatoside C on cell proliferation, cell cycle of HCC cell lines, and Hep3B xenograft model. (**A**) Structure of lanatoside C. (**B**) Hep3B and HA22T cells were exposed to lanatoside C for 48 h, and then detected % of control cell growth by SRB assay. Lanatoside C induced Hep3B cell apoptosis in a concentration-dependent (**C**) and time-dependent (**D**) manner by FACScan flow cytometry analysis with propidium iodide (PI) staining. (**E**) Hep3B cells were incubated with lanatoside C in indicated concentration for 18 hr. Cells were stained with TUNEL assay (green fluorescence) and DAPI (blue fluorescence) in the same area. Magnification of TUNEL staining was 200X. (**F**) SCID mice were ectopically implanted with Hep3B cells. The upper curves show the effect of lanatoside C (2.5 mg/kg, ip, q3d or 2.5 mg/kg, ip, twice a week) on tumor volume and percentage of tumor growth delay (TGD), which was calculated for treatment groups relative to control group; the lower curves show the body weight of mice after indicated treatment. Data are expressed as means ± SEM of three independent determinations. *P < 0.05 and **P < 0.01, untreated cell versus lanatoside C-treated groups.

Lanatoside C inhibited the proliferation of two different human HCC cell lines, Hep3B and HA22T, in a concentration-dependent manner, exhibiting 50% growth-inhibitory concentrations (GI_50_) of 0.12 and 0.14 μM, respectively, in sulforhodamine B assays (Fig. 1B). We next determined the effect of lanatoside C on cell-cycle progression in asynchronized Hep3B cells by flow cytometry analysis of propidium iodide (PI)-stained cells using a FACScan system. Lanatoside C induced a concentration- and time-dependent increase in the percentage of cells in a sub-G_1_ phase (Fig. 1C and D), indicative of apoptotic cells, also shown in other HCC cell lines (Supplementary Fig. S1A). We also examined the effect of lanatoside C on Hep3B cells by terminal deoxynucleotidyl transferase dUTP nick-end labeling (TUNEL) staining (Fig. 1E). We found that lanatoside C caused DNA fragmentation in a concentration-dependent manner in Hep3B cells. In addition, we also evaluated *in vivo* antitumor efficacy of lanatoside C. The results suggest that lanatoside C dramatically decreased tumor volume and delayed tumor growth without obvious body weight loss (Fig. 1F). These results indicate that lanatoside C is capable of inhibiting proliferation and inducing apoptosis in HCC cells.

### Effect of lanatoside C on MMP and mitochondrial-related proteins

**Figure 2:**
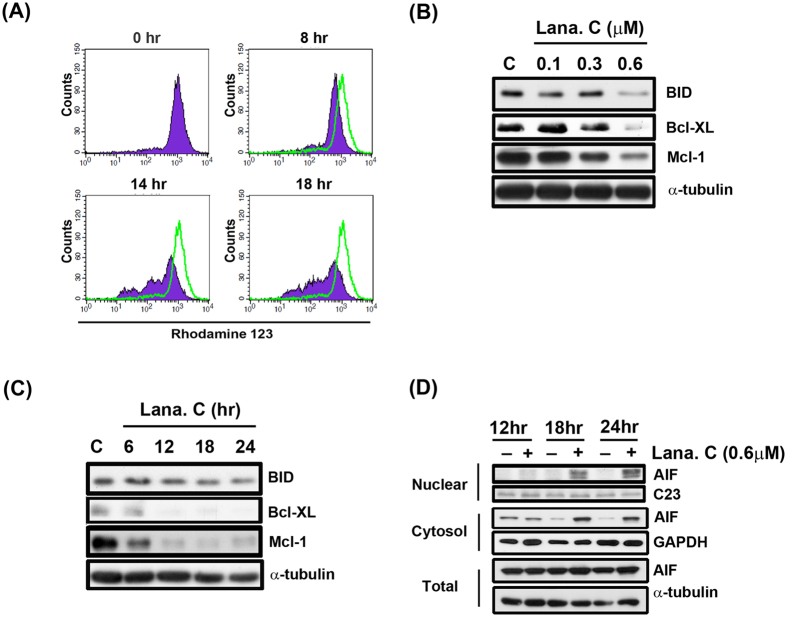
Effect of lanatoside C on MMP and mitochondrial related proteins. (**A**) Hep3B cells were incubated with DMSO (0 h) or with lanatoside C (0.6 μM) at indicated times and then cells were incubated with rhodamine123 (5 μM) for 30 minutes. Data acquisition and analysis were performed on a FACScan flow cytometry. (**B**) Hep3B cells were treated with a range of lanatoside C concentration (0.1–0.6 μM) for 18 hr. (**C**) Hep3B cells were treated with lanatoside C (0.6 μM) for indicated time (6–24 h), and then cells were harvested from total lysates for detection of BID, Bcl-xL and Mcl-1 protein expressions by using Western blot analysis. α-tubulin was used as internal control. (**D**) Hep3B cells were treated lanatoside C (0.6 μM) for 12 h, 18 h, and 24 h and detected of AIF in nuclear, cytosol fraction and total lysate by Western blot analysis. C23, GAPDH and α-tubulin was a nuclear, cytosol and total lysate marker protein, respectively, used as internal controls. Data are representative of three independent experiments.

Loss of MMP is associated with opening of the mitochondrial permeability transition pore, leakage of inner mitochondrial components into the cytosol and subsequent apoptosis^16^. To investigate the effect of lanatoside C on MMP, we incubated Hep3B cells with 0.6 μM lanatoside C for the indicated times and then measured rhodamine 123 fluorescence. As shown in Fig. 2A, lanatoside C induced a significant and sustained reduction in MMP after 14–18 h of treatment. These results indicate that lanatoside C induces mitochondrial dysfunction in Hep3B cells in a time-dependent manner. The Bcl-2 protein family regulate mitochondrial outer membrane permeability, which leads to the intrinsic apoptotic pathway^17^. We found that lanatoside C downregulated the expression of the Bcl-2 family members, BID, Bcl-2 and Mcl-1, in a concentration- and time-dependent manner (Fig. 2B and C).

**Figure 3:**
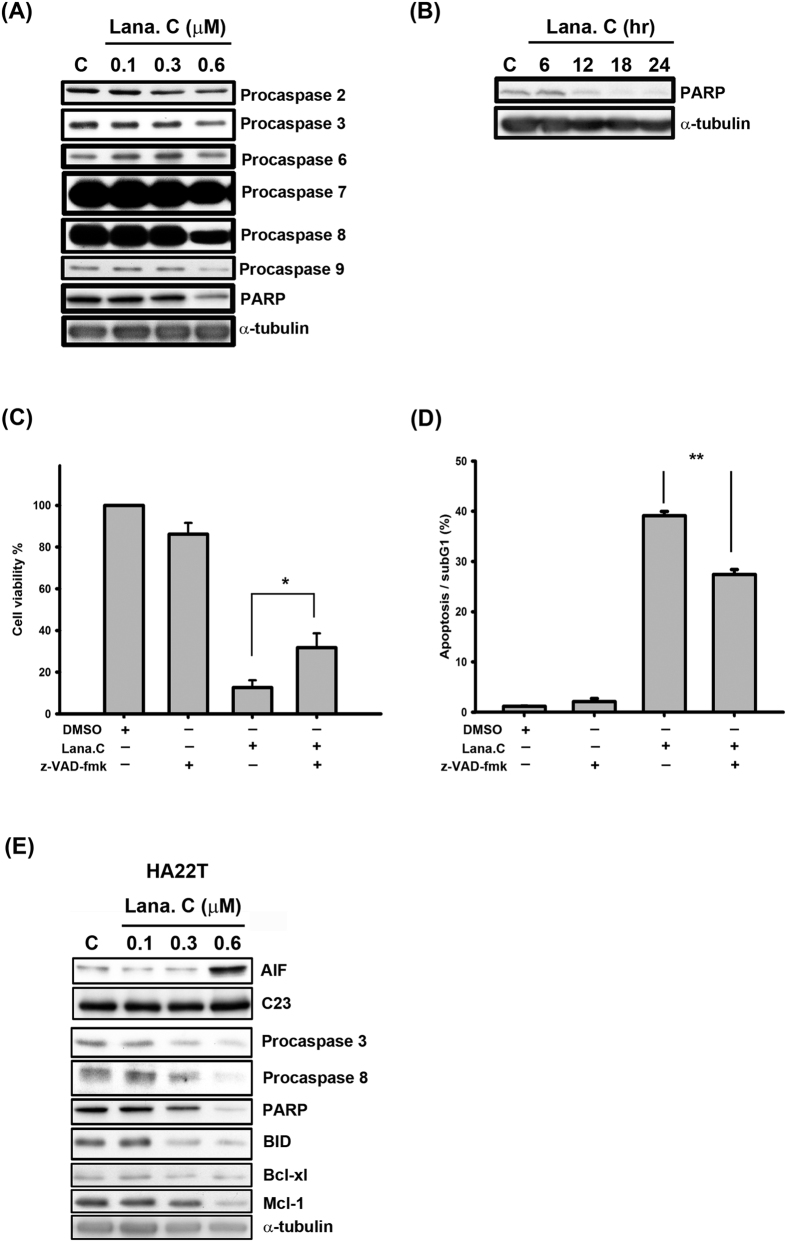
Lanatoside C induced apoptosis in HCC cells. (**A**) Hep3B cells were treated with the indicated concentrations of lanatoside C (0.1–0.6 μM) for 18 h and detected of procaspase-2, -3, -6, -7, -8, -9 and PARP protein expressions by using Western blot analysis. (**B**) Hep3B cells were treated with lanatoside C (0.6 μM) for the indicated time (6–24 h), and cells were harvested from total lysates for detection of PARP protein expressions by using Western blot analysis. Data are representative of three independent experiments. (**C** and **D**) Hep3B cells were incubated in 0.6 μM lanatoside C with or without 100 μM z-VAD-fmk for 24 h. (**C**) The cell viability was determined by using MTT assay as described in methods. Data are repeated at least three independent determinations. *P < 0.05. (**D**) The apoptotic cells were stained with PI and analyzed by flow cytometry as described in methods. Data are repeated at least three independent determinations. **P < 0.01. (**E**) HA22T cells were treated with lanatoside C for 18 h to detect the expressions of caspases and mitochondrial proteins.

Mitochondrial outer membrane permeabilization allows soluble proteins to diffuse into the cytosol from the intermembrane space of mitochondria. One such soluble protein is apoptosis-inducing factor (AIF), which translocates into the nucleus to trigger caspase-independent apoptosis^18^. Our results indicate that lanatoside C markedly increased the amount of AIF in the nuclear fraction in a time-dependent manner (Fig. 2D). Similar results were obtained in HA22T cells, suggesting that lanatoside C causes caspase-independent apoptosis in human HCC cells (Fig. 3E and Supplementary Fig. S2A).

### Lanatoside C induces caspase-dependent apoptosis in HCC cells

Extending these results, we further investigated the effect of lanatoside C on caspase activation, which has a major role in the apoptosis pathway. We found that lanatoside C decreased the proform of initiator caspases (caspase-2, -8 and -9) and effector caspases (caspas-3, -6 and -7) in a concentration-dependent manner (Fig. 3A). Expression of the proform poly (ADP ribose) polymerase (PARP), a downstream substrate of effector caspases, was decreased in lanatoside C-treated Hep3B cells (Fig. 3A and B). In addition, zVAD-fmk, a broad-spectrum caspase inhibitor, reversed the apoptotic effects of lanatoside C (Fig. 3C,D and Supplementary Fig. S2B). Lanatoside C also caused activation of caspase-3, -8 and PARP cleavage in a concentration-dependent manner in HA22T cells (Fig. 3E). Taken together, these results support the conclusion that lanatoside C induces caspase-dependent apoptosis in human HCC cells.

### Lanatoside C triggers PKCδ activation in human HCC cells

**Figure 4:**
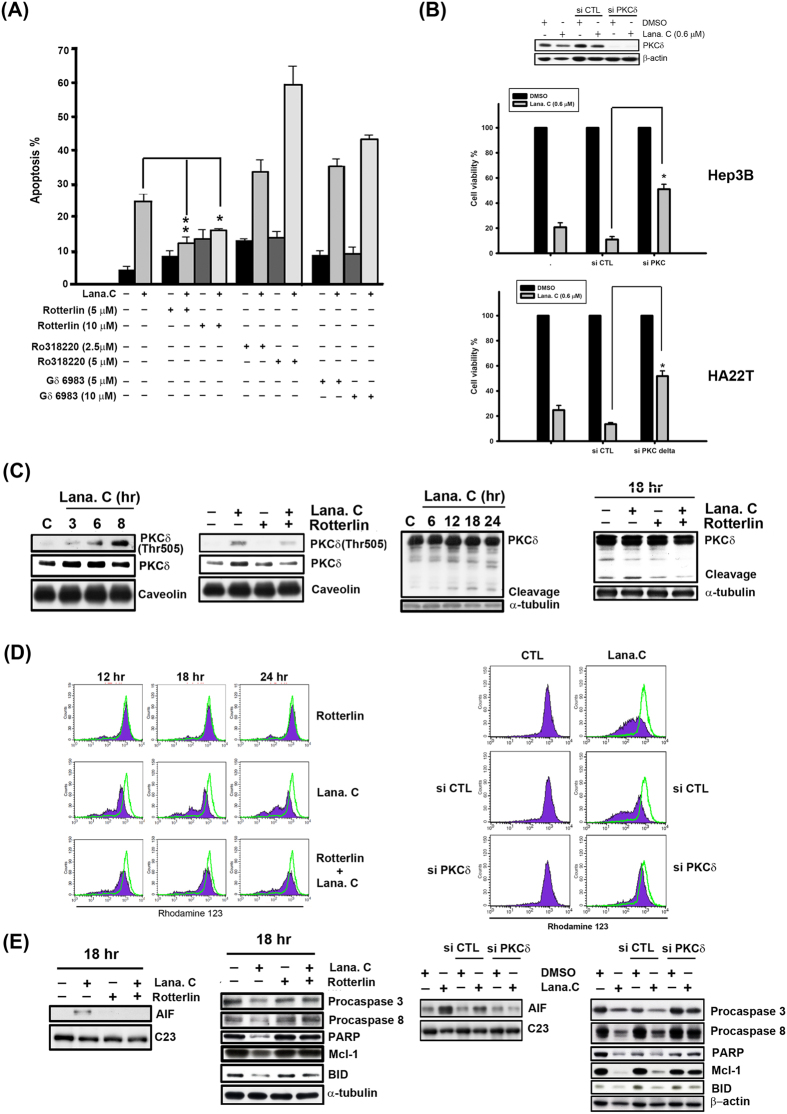
Activation of PKCδ was involved in lanatoside C-induced apoptosis. (**A**) Hep3B cells were incubated with different PKC inhibitors (Ro318220: pan-PKC inhibitor, Gö 6983: classical PKC inhibitor, and Rottlerin: PKCδ inhibitor) for 18 h and subsequently analyzed by FACScan flow cytometry with PI staining to determine their sub-G1 proportion. Data are expressed as means ± SEM of three independent determinations. *P < 0.01 and **P < 0.05, untreated cell versus lanatoside C-treated cells. (**B**) Hep3B and HA22T cells were transfected with PKCδ or control siRNA, followed by treatment with lanatoside C (0.6 μM) for 18 h. Cell viability was determined by MTT assay. (**C**) Hep3B cells were treated with lanatoside C (0.6 μM) or co-incubated with rottlerin (5 μM) for the indicated time and detected of PKCδ Thr505 in membrane fraction and PKCδ from total lysates by Western blot analysis. Caveolin was a membrane marker protein and α-tubulin used as internal control. (**D**) Hep3B cells were treated with lanatoside C (0.6 μM), rottlerin (left panel) or PKCδ siRNA (right panel), or combination treatment for 18 h and then incubated with rhodamine123 (5 μM) for 30 min. Data acquisition and analysis were performed on a FACScan flow cytometry. (**E**) Hep3B Cells were incubated with Lanatoside C (0.6 μM), rottlerin (5 μM) or PKCδ siRNA, or combination treatment for 18 h. Cells were harvested from nuclear fraction and total lysates for detection of the indicated protein expressions by using Western blot analysis. C23 was a nuclear marker protein used as internal control. Results are representative of three independent experiments.

In a previous study, bufalin, a cardiac glycoside, was shown to simultaneously induce cell differentiation and apoptosis through cPKC and PKCδ in human monocytic leukemia THP-1 cells^19^. These PKC isozymes are involved in diverse functions, including induction of cell apoptosis, reflecting differences in their tissue distribution, subcellular localization and substrate specificity^20^. We co-treated Hep3B cells with lanatoside C and different PKC inhibitors, including the pan-PKC inhibitor Ro318220, the cPKC inhibitor Gö6983 or the PKCδ-specific inhibitor rottlerin, and assessed proliferation using MTT assay. These experiments showed that only rottlerin significantly reduced lanatoside C-induced inhibition of Hep3B cell proliferation and did so in a concentration-dependent manner (Fig. 4A). Consistent with this, small interfering RNA (siRNA) targeting PKCδ (siPKCδ) also markedly reversed lanatoside C-induced inhibition of Hep3B and HA22T cell proliferation (Fig. 4B).

It has been demonstrated that the PKB kinase PDK1 is responsible for phosphorylation of Thr505 in the activation loop of PKCδ^21^. Furthermore, the downstream signal, diacylglycerol, acts through the regulatory region of nPKCs to mediate their recruitment to the plasma membrane, which is considered the primary event for nPKC activation^22^. Typically, membrane recruitment alters the conformation of cPKCs and nPKCs, thereby enabling both enzyme activation and subsequent substrate phosphorylation. The plasma membrane fractionation studies showed cells treated with lanatoside C for 3 h increased recruitment of PKCδ to the plasma membrane and induced phosphorylation of activation loop-residue Thr505 in PKCδ at the plasma membrane in Hep3B cells (Fig. 4C). Rottlerin notably inhibited lanatoside C-induced PKCδ phosphorylation and translocation in Hep3B and HA22T cells (Fig. 4C and Supplementary Fig. S2D). A previous study showed that PKCδ is an endogenous substrate for caspase-3 and is cleaved to yield a 40-kDa catalytically activate fragment that leads to apoptosis^23^. As shown in Fig. 4C and Supplementary Fig. S2D, PKCδ was cleaved after treatment with lanatoside C for 18 h in Hep3B and HA22T cells. In contrast, the apoptotic effect is reduced with co-treatment of rotterlin and lanatoside C (Fig. 4E and Supplementary Fig. S2E). Collectively, these results indicate that lanatoside C first induces PKCδ phosphorylation at Thr505 and translocation to the plasma membrane (3–6 h) in HCC cells, and then causes PKCδ cleavage, leading to apoptosis.

### PKCδ is involved in lanatoside C-induced loss of MMP and modulation of mitochondrial-related proteins and caspases

To further determine whether PKCδ plays a role in the lanatoside C-induced apoptotic pathway, we co-treated Hep3B cells with lanatoside C, rottlerin or siPKCδ. As shown in Fig. 4D and E, both rottlerin and siPKCδ markedly inhibited lanatoside C-induced MMP loss, Mcl-1 downregulation, BID proform cleavage, caspase-3 and -8 activation, PARP cleavage, and AIF translocation. Similar results were also shown in HA22T cells (Supplementary Fig. S2A,C and E). Notably, the decrease in cleaved caspase 3 levels after inhibition of PKCδ implies that PKCδ may also act upstream of caspase-3, suggesting the existence of a positive regulatory loop between PKCδ and caspase-3^24^. Collectively, these results show that PKCδ triggers lanatoside C-induced apoptosis via both caspase-dependent and caspase-independent pathways.

### Effect of lanatoside C on the AKT/mTOR pathway

**Figure 5:**
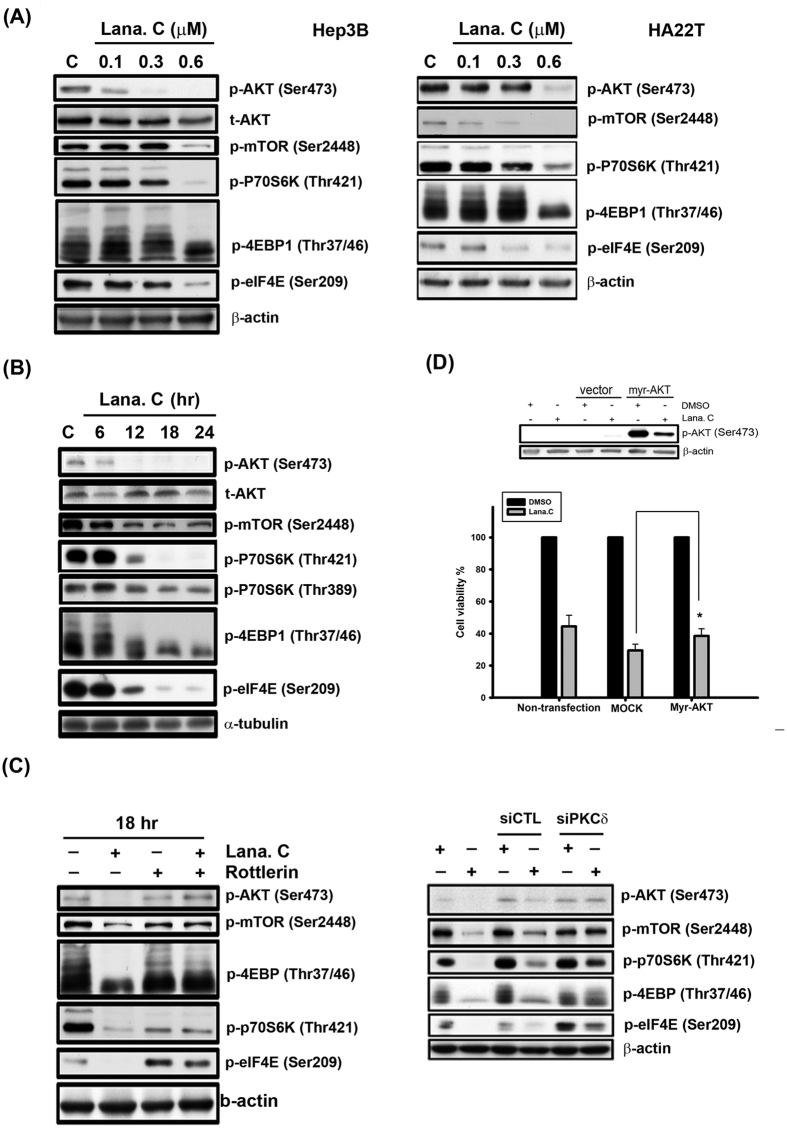
Effect of lanatoside C on AKT/mTOR pathway. (**A**) Hep3B and HA22T cells were treated with a range of lanatoside C (0.1–0.6 μM) for 18 h. (**B**) Hep3B cells were treated lanatoside C (0.6 μM) for indicated time (6–24 hr) and then cells were harvested from total lysates for observation of AKT/mTOR and their downstream signaling protein expressions by using Western blot analysis. (**C**) Hep3B cells were incubated with Lanatoside C (0.6 μM), rottlerin (5 μM) or PKCδ siRNA, or combination treatment for 18 h and then cells were harvested from total lysates for detection of indicated protein expressions by using Western blot analysis. (**D**) Hep3B cells were transfected with empty vector (MOCK) or Myr-AKT for 6 h and re-serum overnight, followed by treatment with or without lanatoside C (0.6 μM) for 18 h. Cells were harvested from total lysates for detection of phospho-AKT Ser473 protein expressions by using Western blot analysis. Cell viability was measured by MTT assay. Data are expressed as means ± SEM of three independent determinations. *P < 0.01, Myr-AKT-overexpressed cells versus MOCK-transfected cells.

Phosphoinositide 3-kinases (PI3Ks) transduce signals from various growth factors and cytokines into intracellular messages by generating phospholipids, which activate the serine-threonine protein kinase Akt; moreover, dual inhibitors of PI3K/mTOR exert strong anti-proliferative activity against tumors^25^. Lanatoside C markedly reduced phosphorylation of Akt and its downstream effectors as well as mTOR and its downstream effectors, such as the mTOR-activated kinase p70S6K and the eukaryotic translation initiation factors 4EBP and eIF4E, in a concentration-dependent manner in both Hep3B and HA22T cells (Fig. 5A). As shown in Fig. 5B, lanatoside C inhibited these kinases in a time-dependent manner (6–24 h) in Hep3B cells. These results also inspired us to explore the relationship between the Akt/mTOR pathway and PKCδ in the context of lanatoside C treatment. Remarkably, we found that both rottlerin and siPKCδ abolished the effects of lanatoside C on the Akt/mTOR pathway (Fig. 5C and Supplementary Fig. S4B). These results strongly suggest that PKCδ regulates the Akt/mTOR pathway. Moreover, we tested the effect of enhancing AKT phosphorylation activity by transiently transfecting Hep3B or HA22T cells with Myc-tagged Akt, and found that overexpression of Akt significantly inhibited lanatoside C-induced cell death (Fig. 4D and Supplementary Fig. S4A). Collectively, these results strongly suggest that lanatoside C suppresses the Akt/mTOR signaling pathway through regulation of PKCδ, leading to enhanced apoptosis in human HCC cells.

### Effect of lanatoside C on the phosphorylation of MAPK kinases

Recent studies have demonstrated a relationship among cardiac glycosides, PKCδ, and mitogen-activated protein kinases (MAPKs)^26^,^27^. Our results showed that lanatoside C decreased phosphorylation of the MAPK, ERK1/2 (extracellular signal-regulated kinase 1/2), but not that of c-Jun N-terminal kinase (JNK) or p38, in Hep3B and HA22T cells, and did so in a concentration-dependent manner; it also exerted a sustained, time-dependent inhibitory effect on ERK1/2 phosphorylation in Hep3B cells (Supplementary Fig. S3A and B). The effect of lanatoside C on ERK1/2 was not blocked by rottlerin, suggesting that PKCδ is not involved in ERK1/2 dephosphorylation (Supplementary Figs S3C and S4B). To confirm the involvement of ERK1/2 inhibition in lanatoside C-induced apoptosis, we tested the effect of enhancing ERK1/2 phosphorylation by overexpressing an HA-tagged version of the ERK1/2 upstream kinase, MEK (MEK-HA), in Hep3B and HA22T cells. As shown in Supplementary Figs S3D and S4A, cell viability was significantly enhanced by transiently overexpressing MEK-HA compared with transfection with a vector control. These results imply that lanatoside C inhibits the ERK1/2 signaling pathway independent of PKCδ activation to enhance apoptosis in HCC cells.

## Discussion

**Figure 6:**
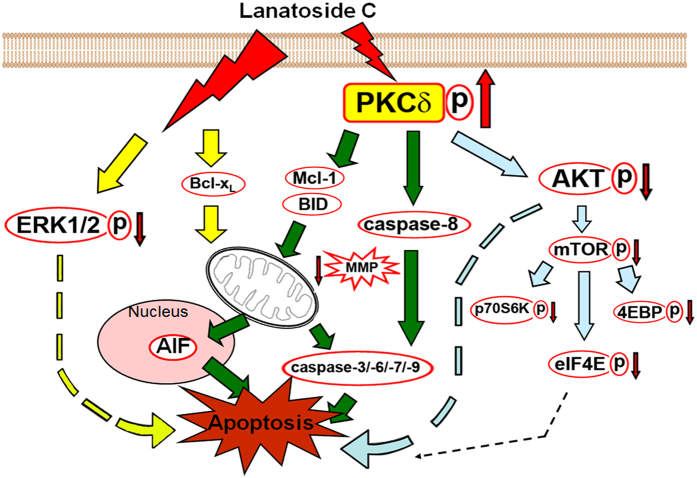
Schematic summary of lanatoside C-triggered apoptosis in human HCC cells by PKCδ activation.

Epidemiological studies have reported that cancer patients receiving digitalis have significantly lower mortality rates, renewing interest in the anticancer properties of cardiac glycosides^28^. *In vitro* and *in vivo* studies conducted over the last 10 years have supported these observations and many clinical trials of cardiac glycosides, or their derivatives as cancer treatments, have been conducted^29^. Numerous studies have reported that digitoxin and digoxin have anticancer effects^30^. The results of these studies have revealed significant differences in cytotoxicity among cardiac glycosides in terms of both potency and selectivity and revealed that the mechanisms underlying their cytotoxicity differ from those of commonly used anticancer drugs^6^. Digoxin, ouabain, and proscillaridin A were shown to attenuate the proliferation of breast cancer MCF-7 cells by inhibiting DNA topoisomerase activity^10^. Oleandrin sensitizes lung cancer cells to Apo2L/TRAIL-mediated apoptosis through upregulation of death receptors-4 and -5 at both RNA and protein levels^31^. In the current study, we showed that lanatoside C, a digitalis derivative, potently inhibits the growth of the HCC cells *in vitro* and *in vivo*. Notably, we provide the first description of the anticancer mechanism of lanatoside C, showing that it induces HCC cell apoptosis mainly through PKCδ activation and partially by inhibiting activation of AKT and ERK (Fig. 6).

It has been reported that cPKC and nPKC are involved in signal transduction events that lead to bufalin-induced cell differentiation and apoptosis, respectively, in human monocytic leukemia THP-1 cells^32^. Therefore, we suggest that lanatoside C-mediated apoptosis is related to the distinct PKC isozymes that direct the fate of individual cells. Etoposide^33^, Ara-C^34^, cisplatin, and methylglyoxal^35^ have been reported to induce apoptosis by activating PKCδ in various cancer cell lines. These findings reveal that PKCδ plays an essential role in the regulation of apoptosis in response to a large and diverse array of apoptotic stimuli. Rottlerin, a PKCδ-specific inhibitor and siPKCδ significantly attenuated lanatoside C-induced apoptosis, suggesting the involvement of PKCδ in this phenomenon (Fig. 4A and B). In contrast, Ro318220 and Gö6983 significantly augmented lanatoside C-induced apoptosis in Hep3B cells (Fig. 4A), implying that no other PKC subtypes are involved. Recent studies have demonstrated that the first phosphorylation in a sequential series of priming phosphorylation events for PKC activation occurs at an ‘activation-loop’ threonine and plays a critical role in aligning residues in the catalytic pocket^36^,^37^. The catalytic activity of membrane-associated, allosterically activated PKCδ is increased by Thr505 phosphorylation, which is mediated by PDK-1, PKCε, Src, or by PKCδ itself^38^. PKCδ activation requires coordinated phosphorylation and translocation events and both mechanisms must be considered in evaluating the PKCδ signaling pathway. We found that PKCδ markedly translocated to the cell membrane of HCC cells and was phosphorylated at Thr505 after a 3 h treatment with lanatoside C (Fig. 4C and Supplementary Fig. S2D).

PKCδ has emerged as a common mediator of apoptosis in response to many stimuli. However, the precise mechanisms by which PKCδ executes its pro-apoptotic actions may vary considerably, depending on the cell type or the nature of the apoptotic stimulus, among other factors^39^. Most studies highlighted above suggest that the role of PKCδ in the induction of apoptosis involves its caspase-dependent cleavage and the presence of a positive regulatory loop between PKCδ and caspase-3^24^,^40^,^41^. We found that lanatoside C induced caspase-3 activation and PKCδ cleavage; both events were attenuated by rottlerin and siPKCδ (Fig. 4C,E, Supplementary Fig. S2D and E), suggesting the presence of a positive loop between PKCδ and caspase-3 that is dependent on PKCδ activity. PKCδ undergoes caspase-3–mediated cleavage, resulting in the release of the catalytic domain; this domain is constitutively active and translocates to mitochondria, triggering the intrinsic apoptotic pathway. Notably, cells from PKCδ-null mice exhibit defective mitochondrial-driven apoptosis and caspase-3 activation in response to etoposide treatment^42^. Full-length PKCδ can also translocate to the cell membrane to modulate the secretion of tumor necrosis factor (TNF)-α and TRAIL (TNF-related apoptosis-inducing ligand), leading to triggering of the extrinsic apoptotic pathway. Caspase-8 is a crucial mediator of this PKCδ autocrine effect^43^. In our study, rottlerin and siPKCδ inhibited lanatoside C-induced MMP loss and downregulation of Mcl-1 and BID. In addition, rottlerin and siPKCδ significantly attenuated the pro-apoptotic effects of lanatoside C, such as the limited proteolysis of PKCδ, caspase-3, caspase-8, PARP, and increased release of AIF, suggesting a notable involvement of PKCδ in lanatoside C-induced, caspase-dependent apoptosis (Fig. 4D,E, Supplementary Fig. S2A and E). Interestingly, we first found that rottlerin and siPKCδ also significantly inhibited AIF release, suggesting that lanatoside C also induces caspase-independent apoptosis through PKCδ activation.

Many studies have demonstrated that the PI3K/Akt signaling pathway plays a critical role in controlling the balance between cell survival and apoptosis^25^. mTOR is a downstream target of Akt signaling that regulates translation by directly phosphorylating the key translation regulators p70S6K and 4EBP1. Figure 5(A,B and C)shows that lanatoside C treatment downregulated the AKT/mTOR signaling pathway. Consistent with previous reports that PKCδ can positively or negatively regulate AKT activity depending on cell type and conditions^44^,^45^, we further demonstrated a role for AKT in PKCδ-mediated HCC cell apoptosis. In our study, rotterlin and siPKCδ clearly reversed lanatoside C-induced decreases in the expression of AKT/mTOR signaling proteins (Fig. 5C and Supplementary Fig. S4B). Additionally, lanatoside C-induced cytotoxicity was partially attenuated by overexpression of AKT (Fig. 5D and Supplementary Fig. S4A). It has been noted that PKCδ inactivates AKT, either directly or indirectly, by reducing phosphorylation at Ser473 through activation of protein phosphatase 2A to trigger cell apoptosis^44^,^46^; thus, the precise mechanism by which PCKδ suppresses AKT activity in the current context remains to be elucidated.

A previous study showed that cardiac glycosides activate Src/EGFR (epidermal growth factor receptor) and downstream signaling via Ras/Raf/MEK/ERK by inhibiting Na^+^/K^+^-ATPase^47^. Furthermore, PKCδ overexpression has been shown to cause an increase in ERK phosphorylation, thereby enhancing cell proliferation^48^. However, our study found that lanatoside C suppressed ERK activation in a time- and concentration-dependent manner (Supplementary Fig. S3A and B), an observation inconsistent with the mechanism reported for ouabain, digoxin and proscillaridin A, which induced ERK activation^49^. ERK dephosphorylation did indeed partially participate in lanatoside C-triggered apoptosis (Supplementary Fig. S3D and A), but this aspect of lanatoside C’s actions was not regulated by PKCδ (Supplementary Fig. S3C and B).

Taken together, the results of our study reveal the novel finding that lanatoside C, a type of cardiac glycoside extracted from *Digitalis lanata*, significantly induces HCC cell caspase-dependent and -independent apoptosis. Moreover, we characterized the important roles of PKCδ, AKT, and ERK in lanatoside C-induced anticancer effects. It is well documented that, not only are cardiac glycosides highly effective at inhibiting proliferation of malignant cells, but also effectively sensitize tumor cells to irradiation^50^. These results provide a strong rationale for using lanatoside C as an HCC monotherapy or in conjunction with more standard HCC therapies.

## Additional Information

**How to cite this article:** Chao, M.-W. *et al*. Lanatoside C, a cardiac glycoside, acts through protein kinase Cd to cause apoptosis of human hepatocellular carcinoma cells. *Sci. Rep.* **7**, 46134; doi: 10.1038/srep46134 (2017).

**Publisher's note:** Springer Nature remains neutral with regard to jurisdictional claims in published maps and institutional affiliations.

## Supplementary Material

Supplementary Data
