# Supplementary material for: Lanatoside C, a cardiac glycoside, acts through protein kinase Cδ to cause apoptosis of human hepatocellular carcinoma cells
Source: Sci Rep. 2017 Apr 7;7:46134. doi: 10.1038/srep46134 (PMC5384006; doi:10.1038/srep46134)

Lanatoside C, a cardiac glycoside, acts through protein kinase Cδ to cause apoptosis of human hepatocellular carcinoma cells

Min-Wu Chao[1*], Tzu-Hsuan Chen[2*], Han-Li Huang[1*], Yu-Wei Chang[3], Wei-Chun HuangFu[1], Yu-Ching Lee[4,5], Che-Ming Teng[3,6], Shiow-Lin Pan[1,7]

[1] *The Ph.D. Program for Cancer Biology and Drug Discovery, College of Medical Science and Technology, Taipei Medical University*
[2] *Division of Industrial Promotion, Development Center for Biotechnology*
[3] *Pharmacological Institute, College of Medicine, National Taiwan University*
[4] *The Center of Translational Medicine, Taipei Medical University*
[5] *Ph.D. Program for Biotechnology in Medicine, College of Medical Science and Technology, Taipei Medical University*
[6] *School of Pharmacy, College of Pharmacy, Taipei Medical University*
[7] *Department of Pharmacology, College of Medicine, Taipei Medical University*

* equal contribution first author

Correspondences:

Shiow-Lin Pan, Ph.D.
The Ph.D. Program for Cancer Biology and Drug Discovery, College of Medical Science and Technology, Taipei Medical University, No. 250, Wu-hsing St., Taipei 11031, Taiwan.
Tel: 886-2-27361661 # 7671
E-mail: slpan@tmu.edu.tw

Min-Wu Chao: Jaw.fay@gmail.com
Tzu-Hsuan Chen: r91443007@ntu.edu.tw
Han-Li Huang: hanlilijh@gmail.com
Yu-Wei Chang: yahwehmax@hotmail.com
Wei-Chun HuangFu: clarehf@tmu.edu.tw
Yu-Ching Lee: b8404056@tmu.edu.tw
Che-Ming Teng: cmteng@ntu.edu.tw

Shiow-Lin Pan: slpan@tmu.edu.tw

(A)

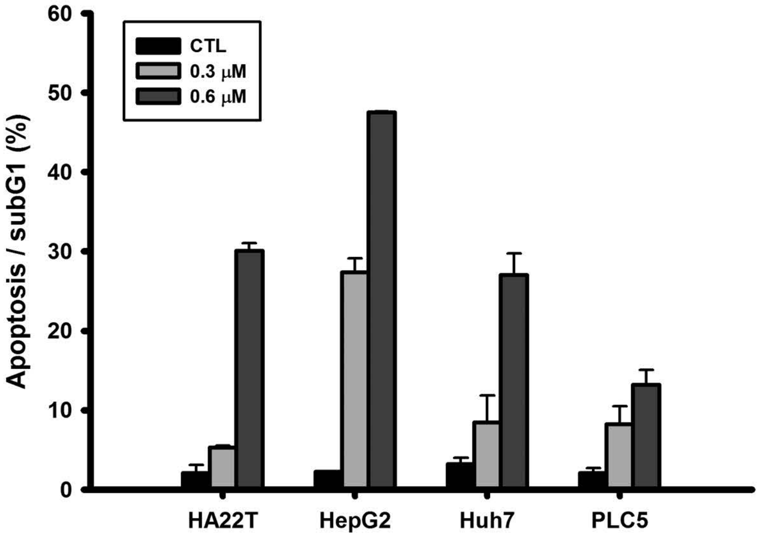

(B)

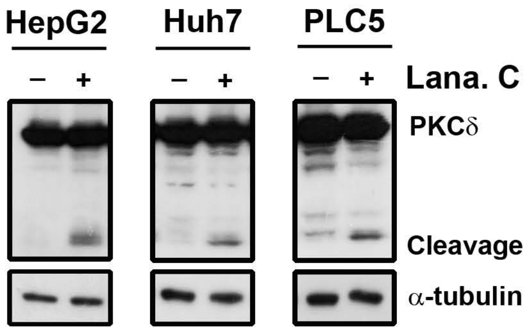

Supplementary Figure S1. Lanatoside C induced apoptosis and PKCδ activation in other HCC cells.

(A) HA22T, HepG2, Huh7 and PLC5 cells were treated with lanatoside C (0.6 μM) for 48 h. Apoptotic cells were stained with PI, and then analyzed by flow cytometry. (B) HepG2, Huh7 and PLC5 cells were incubated with lanatoside C (0.6 μM) for 48 h and PKCδ from total lysates by Western blot analysis.

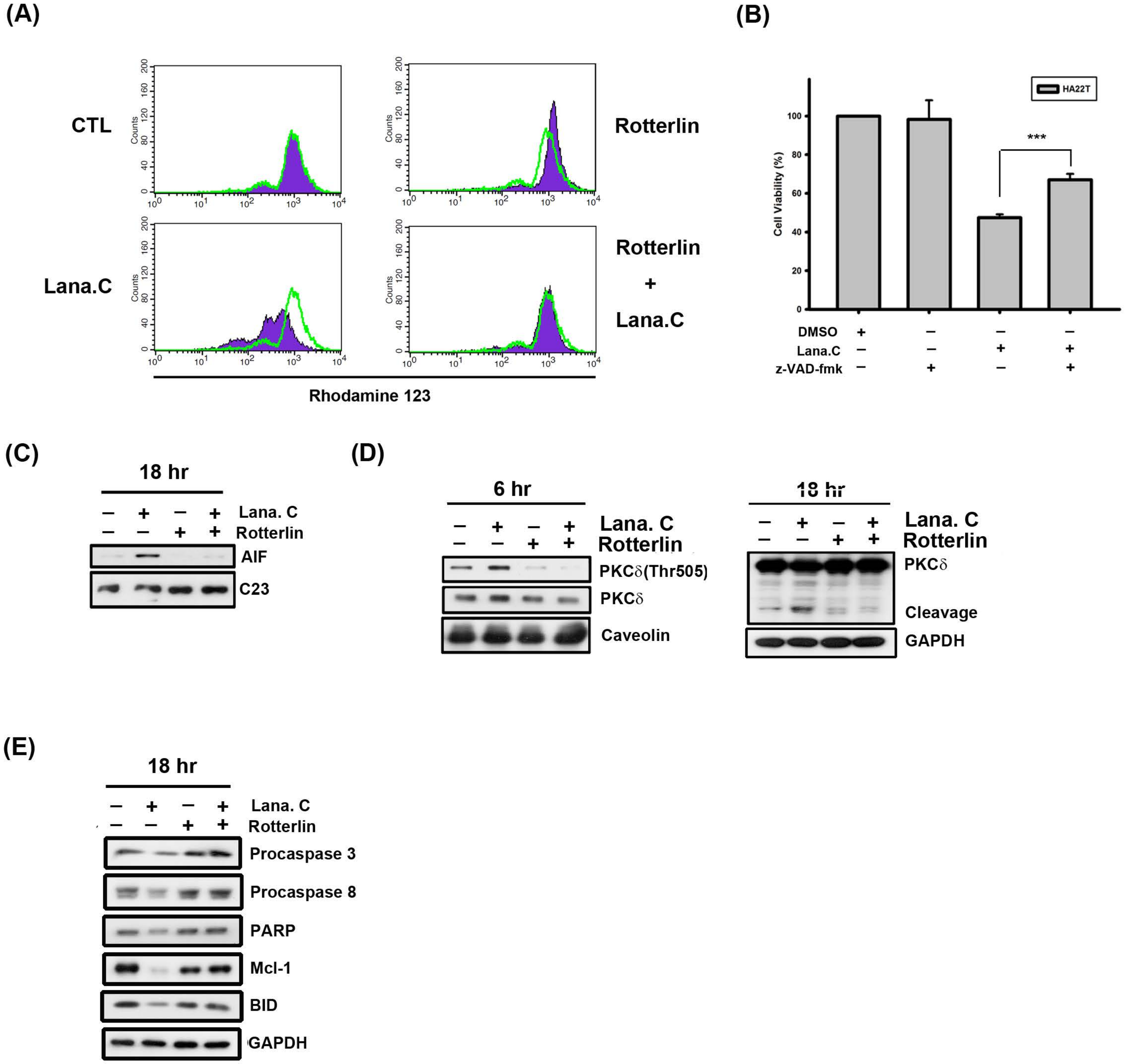

Supplementary Figure S2. Lanatoside C triggered apoptosis was through PKCδ activation in HA22T cell line. (A) HA22T cells were incubated with DMSO or with lanatoside C (0.6 μM), in the presence or absence of rotterlin (10 μM), for 18 h, and then cells were incubated with rhodamine123 (5 μM) for 30 minutes. Data acquisition and analysis were performed on a FACScan flow cytometry. (B) HA22T cells were incubated in 0.6 μM lanatoside C with or without 100 μM z-VAD-fmk for 24 h. The cell viability was determined by using MTT assay as described in methods. Data are repeated at least three independent determinations. *** $P < 0.001$. (C) HA22T cells were incubated with lanatoside C (0.6 μM), rottlerin (10 μM), or combination treatment for 18 h, and then cells were harvested from nuclear fraction for detection of the AIF expression. C23 was a nuclear marker protein used as internal control. (D) HA22T cells were treated lanatoside C (0.6 μM) or co-incubated with rottlerin (10 μM) for 18 h and detected of PKCδ Thr505 in membrane fraction and PKCδ from total lysates by Western blot analysis. Caveolin was a membrane marker protein and GAPDH used as internal control. (E) HA22T cells were incubated with lanatoside C (0.6 μM), rottlerin (10 μM), or combination treatment for 18 h, and then cells were harvested from total lysates for detection of the indicated protein expressions by using Western blot analysis. Results are representative of three independent experiments.

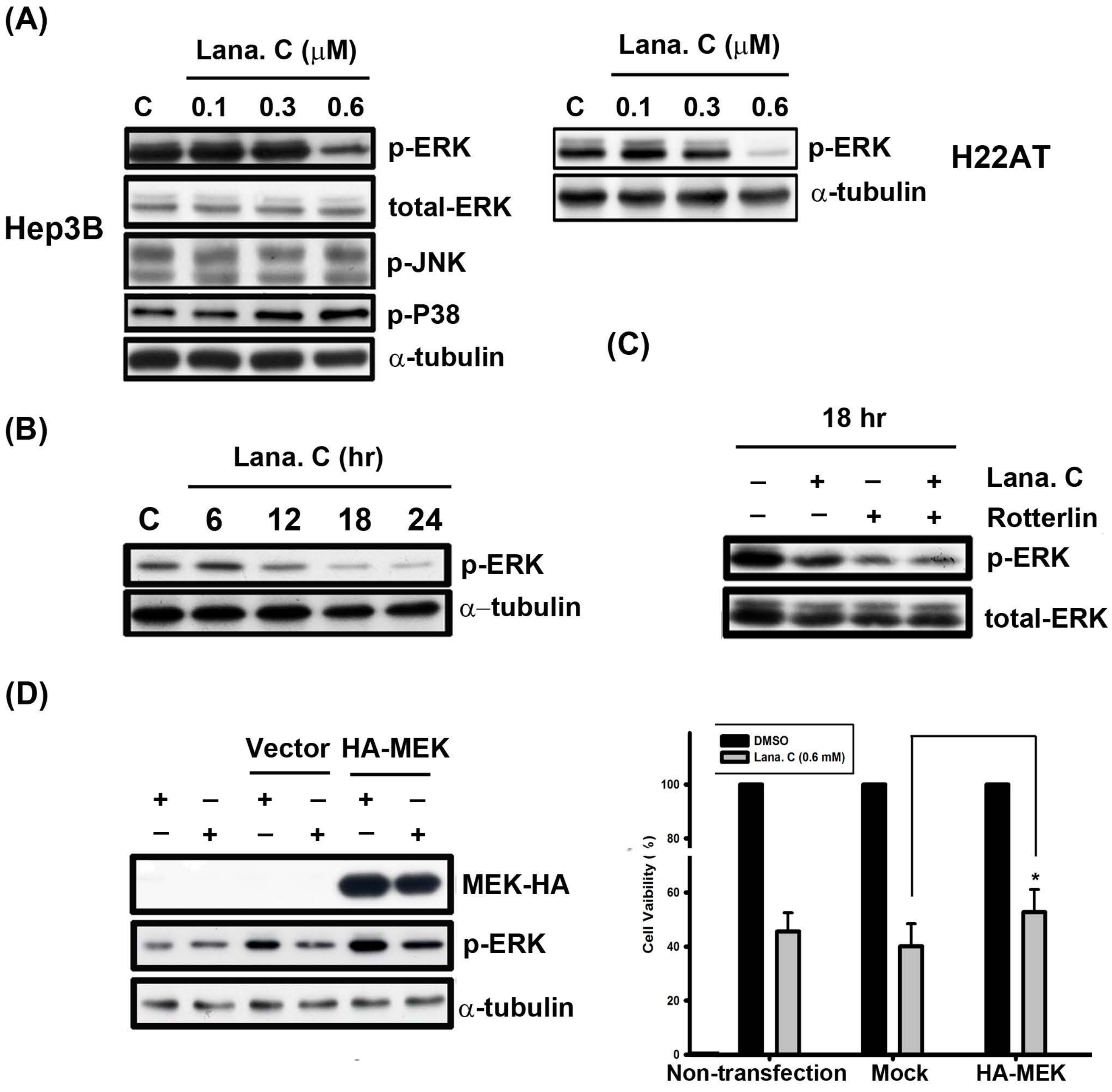

Supplementary Figure S3. Effect of lanatoside C on MAPK pathway.

(A) Hep3B and HA22T cells were treated with a range of lanatoside C (0.1-0.6 μM) for 18 h. (B) Hep3B cells were treated lanatoside C (0.6 μM) for indicated time (6-24 hr), and then cells were harvested from total lysates for observation of p-ERK,total-ERK, p-JNK, and p-P38 by using Western blot analysis. α-tubulin as internal control. (C) Hep3B cells were incubated with lanatoside C (0.6 μM), rottlerin (5 μM) or PKCδ siRNA, or combination treatment for 18 h,and then cells were harvested from total lysates for detection of indicated protein expressions by using Western blot analysis. (D) Hep3B cells were transfected with empty vector (MOCK) or HA-MEK for 6 h, and re-serum overnight, followed by treatment with or without lanatoside C (0.6 μM) for 18 h.Cells were harvested from total lysates for detection of ERK phoshporylation by using Western blot analysis. Cell viability was measured by MTTassay. Data are expressed as means ± SEM of three independent determinations. * $P < 0.01$, HA-MEK-overexpressed cells versus MOCK-transfected cells.

(A)

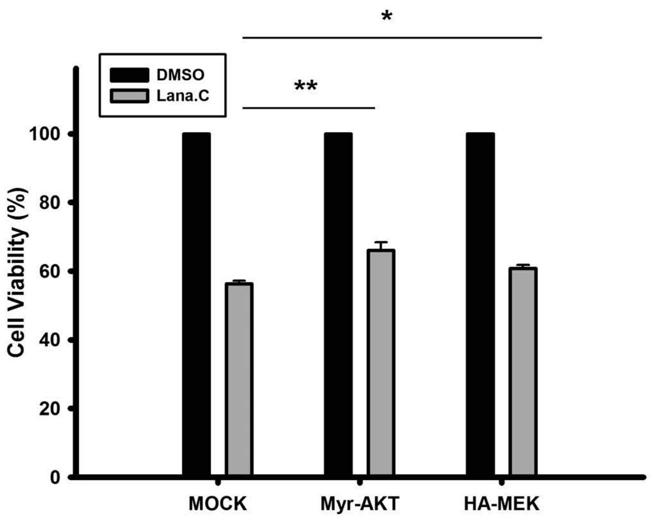

(B)

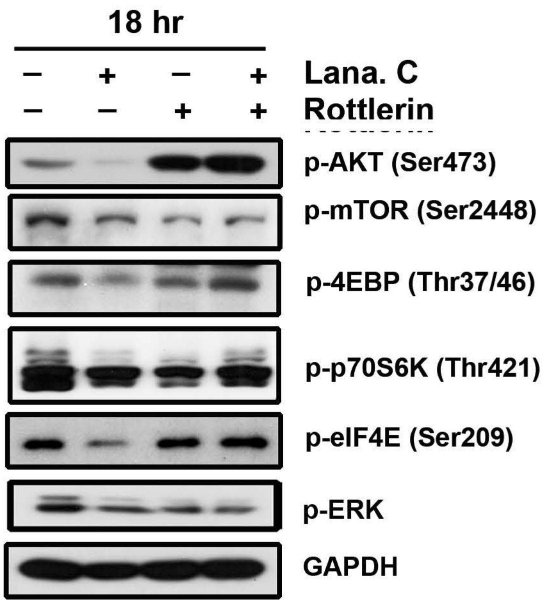

Supplementary Figure S4. PKCδ was involved in lanatoside C induced AKT/mTOR pathway downregulation.

(A) HA22T cells were transfected with empty vector (MOCK) or Myr-AKT or HA-MEK for 8 h, and re-serum overnight, followed by treatment with or without lanatoside C (0.6 μM) for 18 h. Cell viability was measured by MTT assay.

(B) HA22T cells were incubated with lanatoside C (0.6 μM), rottlerin (10 μM) or combination treatment for 18 h, and then cells were harvested from total lysates for detection of indicated protein expressions by using Western blot analysis. assay. Data are expressed as means ± SEM of three independent determinations. Data are expressed as means ± SEM of three independent determinations. * $P < 0.05$, ** $P < 0.01$.

Figure 2

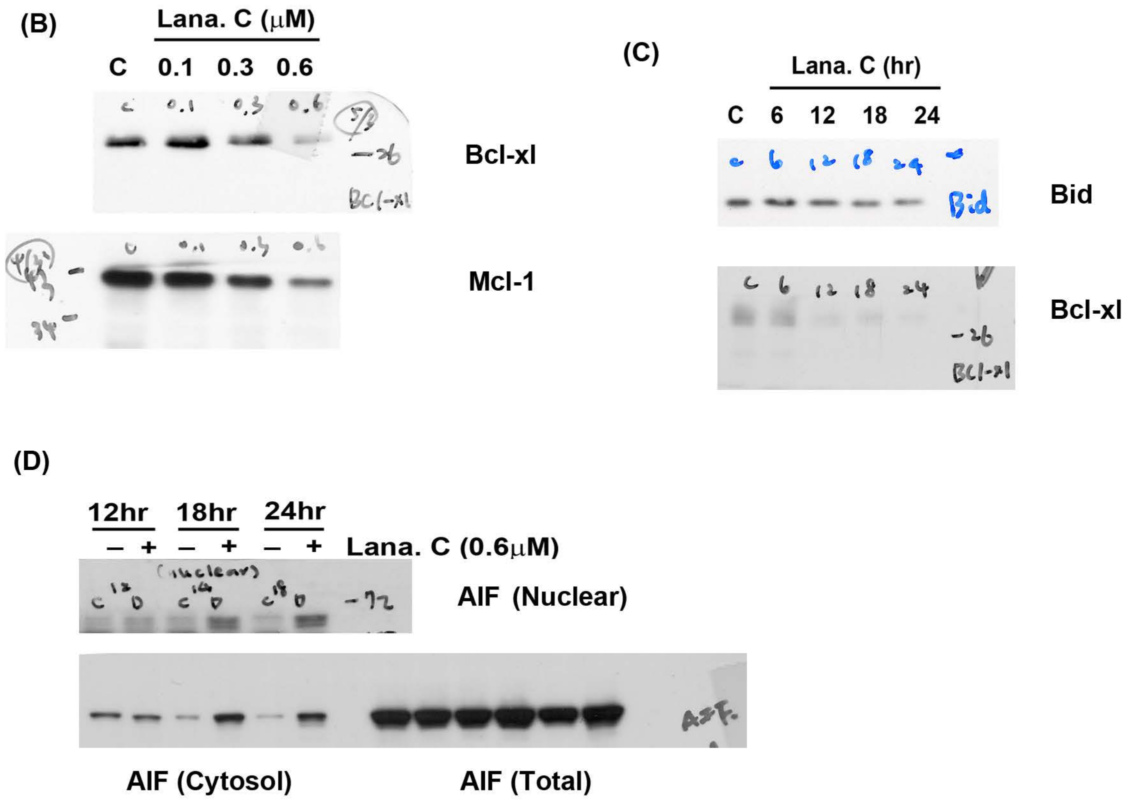

Figure 3

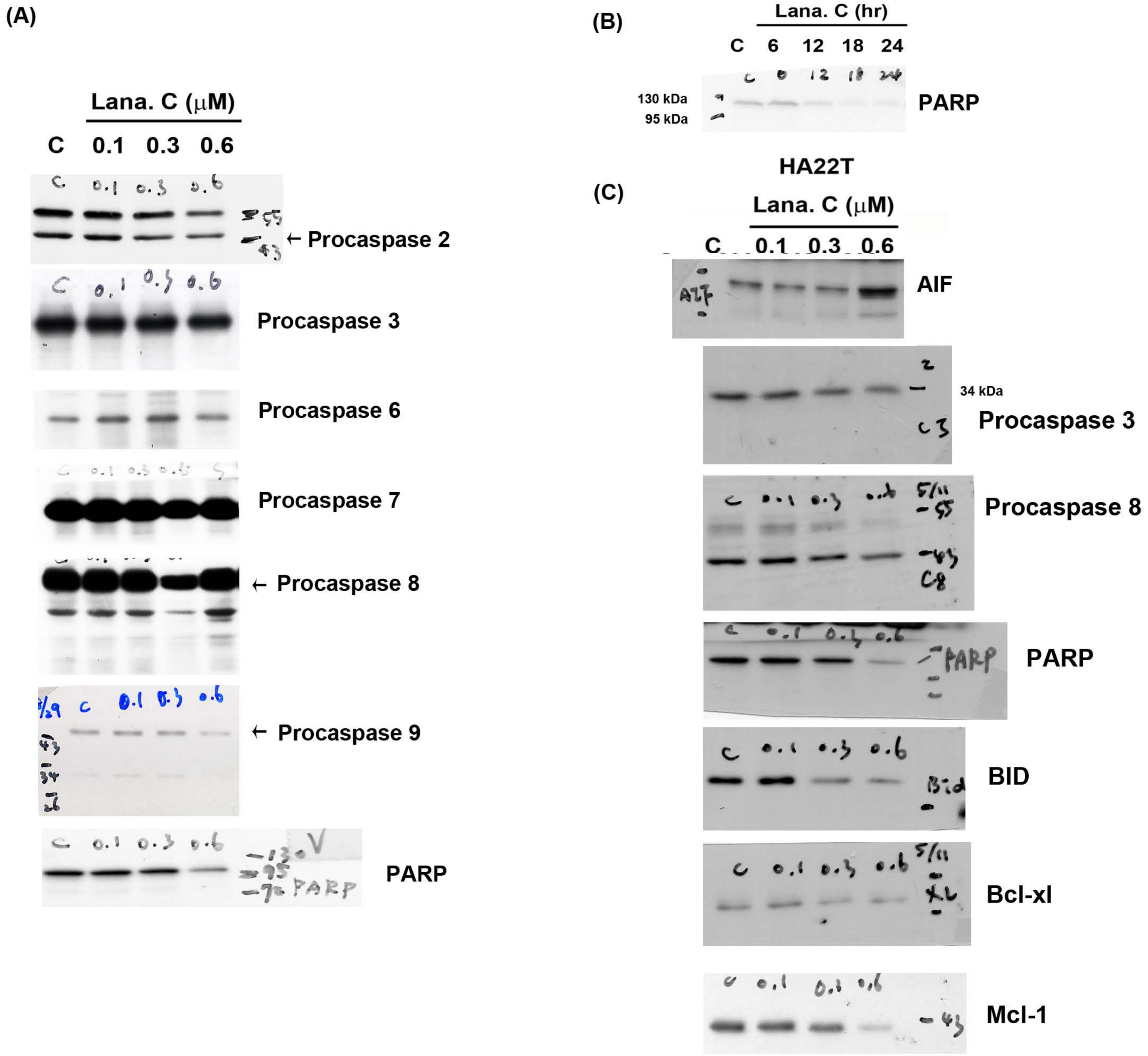

Figure 4.

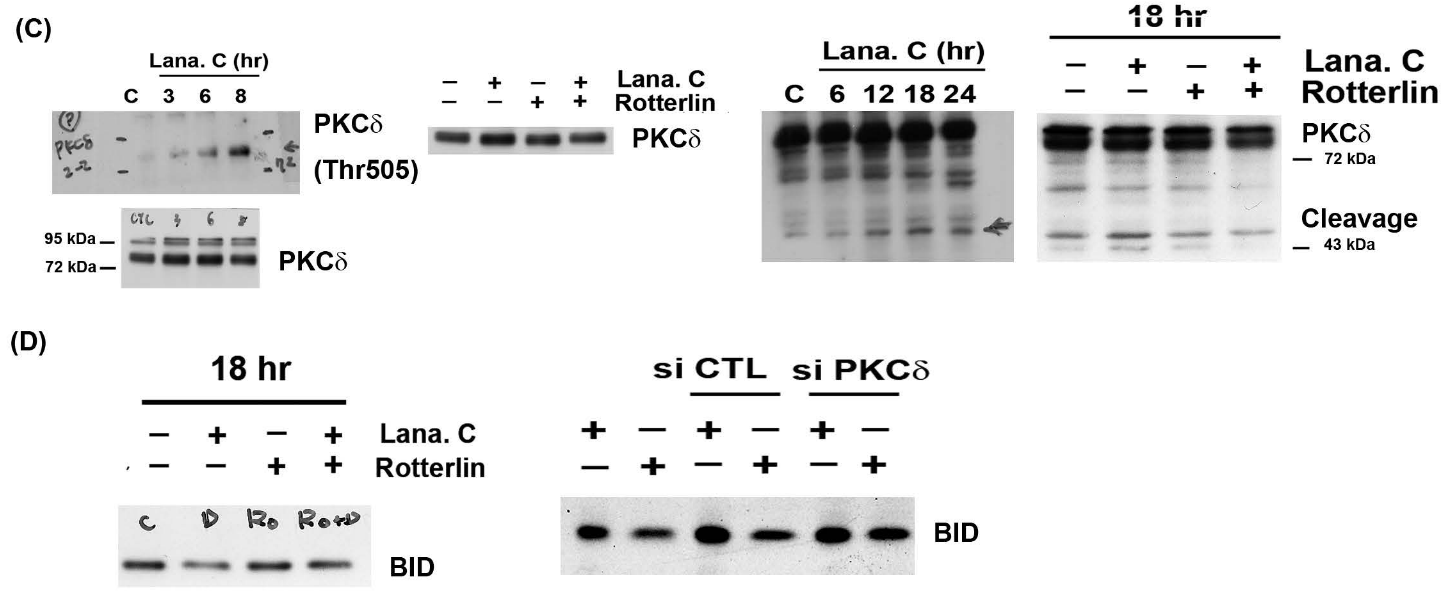

Figure 5.

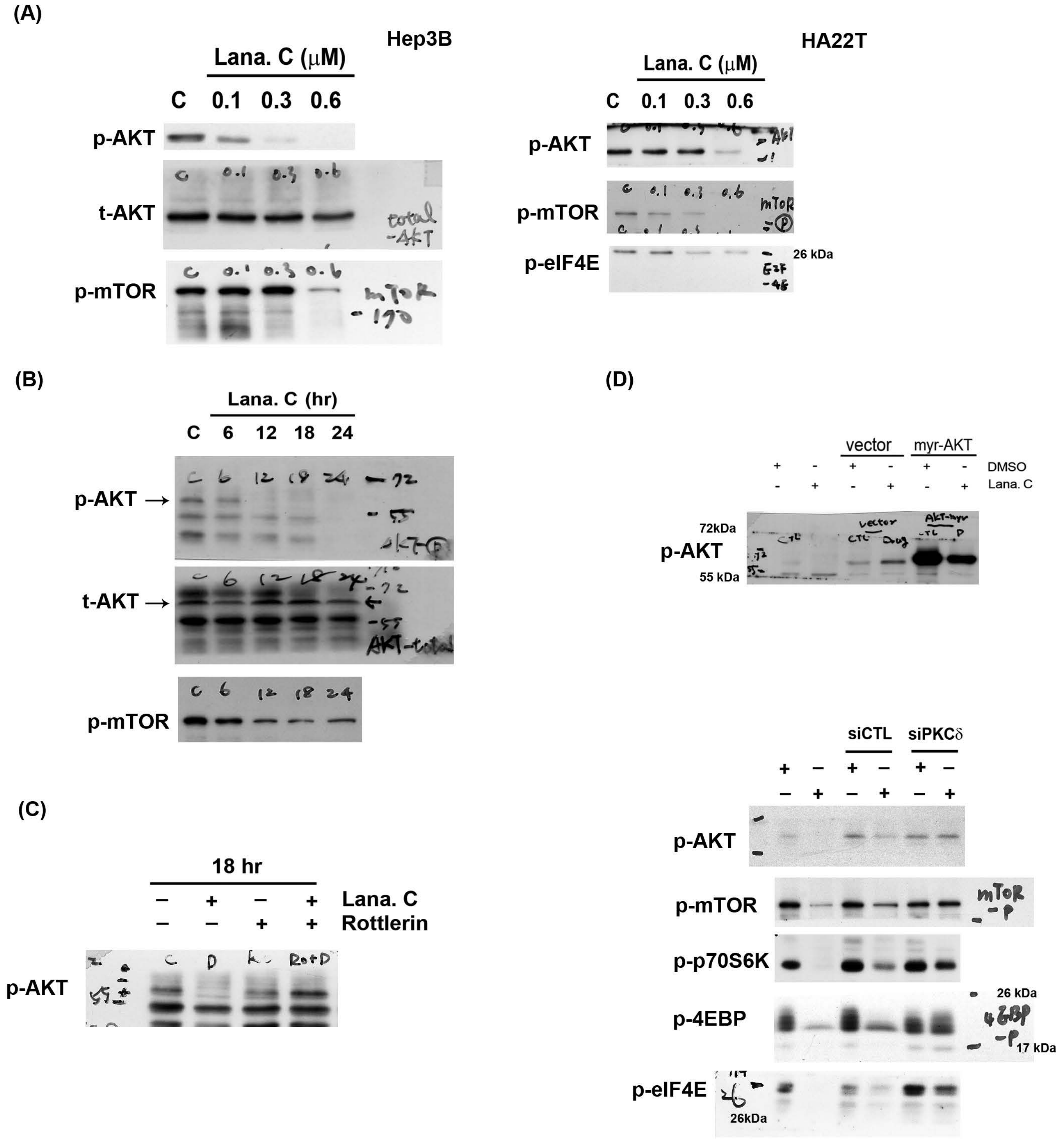

Supplement: Supplementary Data [file srep46134-s1.pdf]
